# Sub-Peritoneal Hæmatocele

**Published:** 1890-01

**Authors:** Eugene Clark

**Affiliations:** Lockhart, Texas


For Daniel's Texas Medical Journal.

### SUB-PERITONEAL HÆMATOCELE.

BY EUGENE CLARK, M. D., LOCKHART, TEXAS.

Read at Austin District Medical Society, September 20, 1889.

THE subject of this paper is a case of Subperitoneal Pelvic Hæmatocele occurring recently in my practice. Thinking it might interest the members of the Society, I take great pleas ure in reporting it. I was called on the morning of July 23rd to visit Mrs. W., who resides 14 miles in the country. She gave the following history: Age 35, began to menstruate at the age of 16 and has been tolerably regular. Married at 20 and has never been pregnant. Has complained more or less with the left side for 15 years. She could lie on that side only a few moments at a time. During the menstrual period, the pain was increased and extended down the left hip and leg. Has suffered greatly with varicose veins in the left leg. Has had no abdominal enlargement previous to present illness. She was taken July 4th with fever. She took calomel on the 4th, 6th and 7th, and was pretty badly salivated. On the 14th was taken with pain in the left groin, which gradually grew more severe and spread over the left side. Her monthly period had come on her just previous to the 14th and returned every two weeks till the 20th of August. She suffered with pains in the pelvic region until the night of the 22nd, when they became excruciating, with cold hands and feet and great irritability of the bladder. When I arrived, the patient was resting quietly under the influence of morphine. I found the pulse quick, temperature normal, the abdomen in the left iliac region enlarged and hard. Upon introducing the finger in the vagina, a hard, bulging mass was encountered, and the uterus was found jammed up close behind the symphysis pubis. I ordered a mouth wash, opiates sufficient to ease and quiet patient. Iodide of potash internally. Blistered the abdomen with iodine, the hot water douche, and enforced absolute rest in the recumbent position. Patient rested quietly and kept quite cheerful till the evening of August 4th, when she was taken with a hard chill, followed by high fever and delirium. I saw her on the 9th; found her with high fever, coated tongue, foul breath, anorexia and profuse sweats. The abdominal enlargement and the mass in the vagina had softened.

Great abdominal tenderness which had been spreading since the chill on the 4th. Well marked fluctuation could be detected by bi-manual palpation between the abdominal enlargement and the mass in the vagina.

I returned on the 10th; the patient was rapidly growing worse.

Pulse quick and temperature high, features pinched, great abdominal tenderness. Being satisfied that the patient was suffering from blood poisoning, the result of absorption of the softened clot, I determined to get rid of it, and aspirated per vaginum and removed 120 oz. of dark grumous fluid, which was odorless and contained some pus. The patient was put on quinine, brandy, beef tea, etc. Absolute rest and the hot water douche continued. On the 11th, the patient was cheerful, temperature normal and pulse good. Some appetite, patient eating with some relish. Pain and tenderness improving.

14th. She has gradually improved. Appetite good. Pain and tenderness disappearing.

20th. Patient able to sit up; had no return of hemorrhage. Pain and tenderness entirely disappeared. Appetite good; has had no fever since the fluid was removed.

At present she is able to be about, and can rest on her left side better than she has done for years.

The source of hemorrhage in this case must have been the rupture of a vein in the pampiniform plexus on the left side where the veins must have been in a varicose condition as shown by the pain and uneasiness, and her inability to lie on that side, all of which has been greatly improved, which improvement must be the result of slight inflamatory action, pressure of the clot and contraction of the sack. The hemorrhage must have been very slow, and lasted from the 14th to the 22nd, the greatest amount taking place on the night of the 22nd, up to which time she had been able to sit up and get about the room. Had the hemorrhage been rapid with such an amount, we would have had dangerous, if not fatal, collapse. The accumulation was undoubtedly subperitoneal; or if in the peritoneal cavity it had become encysted. I am inclined to think the former, although Emmet says that an accumulation in the cellular tissue of the pelvis cannot lift the peritoneum to any great extent without rupture. Where the hemorrhage is in the peritoneal cavity, we are not likely to have the uterus much displaced otherwise than prolapsed, the fluid being able to get in front as well as behind the uterus. Also when the hemorrhage is into the peritoneal cavity it is much more rapid than in the cellular tissue, there being a cavity already prepared in one case, in the other a cavity has to be made and more or less resistance is encountered.

